# Reducing State and Trait Anxiety Through Art Therapy in Adolescents with Eating Disorders: Results from a Pilot Repeated-Measures Study

**DOI:** 10.3390/jcm14155298

**Published:** 2025-07-27

**Authors:** Francesco Monaco, Annarita Vignapiano, Stefania Landi, Ernesta Panarello, Benedetta Di Gruttola, Naomi Gammella, Silvia Adiutori, Eleonora Acierno, Valeria Di Stefano, Ilaria Pullano, Giulio Corrivetti, Luca Steardo Jr

**Affiliations:** 1 Department of Mental Health, ASL Salerno, 84121 Salerno, Italy; f.monaco@aslsalerno.it (F.M.); a.vignapiano@aslsalerno.it (A.V.); n.gammella@aslsalerno.it (N.G.); g.corrivetti@ebris.eu (G.C.); 2 European Biomedical Research Institute of Salerno (EBRIS), 84125 Salerno, Italy; 3 Accademia di Belle Arti, 610074 Roma, Italy; s.adiutori@abaroma.it (S.A.);; 4 Psychiatric Unit, Department of Health Sciences, University Magna Graecia of Catanzaro, 88100 Catanzaro, Italy; valeria.distefano@studenti.unicz.it (V.D.S.); steardo@unicz.it (L.S.J.)

**Keywords:** eating disorders, art therapy, textile art, rehabilitation, adolescents, anxiety

## Abstract

**Background:** Art therapy is increasingly recognized as a valuable complementary intervention for individuals with eating disorders (EDs), who frequently experience comorbid anxiety and difficulties with emotional regulation. However, few studies have examined its short-term effects on state and trait anxiety within structured clinical settings. **Methods:** This pilot study involved 19 adolescent females (mean age 17.7 ± 2.1 years) diagnosed with anorexia nervosa (AN) or bulimia nervosa (BN) and admitted to the Mariconda Regional Residence for Eating Disorders (ASL Salerno, Italy) in residential or semi-residential treatment. Participants completed a structured six-week cycle of weekly textile-based art therapy sessions, designed to promote emotional expression and body reconnection. State and trait anxiety levels were assessed pre- and post-session using the State-Trait Anxiety Inventory (STAI). Repeated-measures ANOVA was used to analyze state anxiety changes; a linear mixed-effects model was applied to trait anxiety. **Results:** State anxiety significantly decreased immediately after sessions (*p* = 0.002). A significant main effect of session (*p* = 0.01) and a time × session interaction (*p* = 0.025) indicated variability across sessions. Trait anxiety showed a non-significant trend toward reduction (*p* = 0.11); however, reductions were significant at sessions 4 (*p* = 0.015), 5 (*p* < 0.001), and 6 (*p* = 0.005). **Conclusions:** Art therapy may offer immediate reductions in state anxiety and may contribute to a longer-term reduction in trait anxiety with 4–6 sessions. These findings support integrating creative interventions within multidisciplinary ED treatment programs. Future research with larger samples and control groups is needed to confirm and expand upon these preliminary results.

## 1. Introduction

Art therapy, a psychotherapeutic technique that employs art media as a primary means of expression and communication, is increasingly recognized as a complementary treatment for mental disorders [1]. The term “art therapy” was first introduced by British artist Adrian Hill in his seminal work *Art versus Illness* (1945), where he described the therapeutic benefits of artistic activity during his recovery from tuberculosis [2]. However, the roots of therapeutic art use can be traced back to the 19th century, when artists and psychiatrists observed the expressive potential of creative works produced by individuals with mental illness [1,3,4]. The systematic development of art therapy as a distinct clinical practice took shape during the mid-20th century in both the United States and Europe [3,5]. In the United States, pioneers such as Margaret Naumburg emphasized the role of spontaneous art expression as a means of accessing unconscious processes, aligning art therapy with psychoanalytic principles [5]. In contrast, Edith Kramer underscored the intrinsic healing properties of the creative process itself, connecting art therapy to developmental psychology and the concept of sublimation [6]. In Europe, figures like Irene Champernowne and Edward Adamson advanced art therapy practices within psychiatric hospitals, contributing significantly to the establishment of professional training programs and the institutional recognition of the discipline [7].

Within contemporary therapeutic contexts, art therapy offers a structured framework that integrates the creative process, applied psychology, and the phenomenology of human experience to promote personal change within a safe and supportive environment facilitated by the therapeutic relationship [1,8,9]. Theoretical orientations in modern art therapy are diverse, ranging from psychodynamic and humanistic approaches to cognitive-behavioral and mindfulness-based models [8,9]. Moreover, neuroscientific research has begun to elucidate the mechanisms underlying art therapy’s efficacy, highlighting its capacity to engage sensorimotor systems, regulate affect, and facilitate the integration of traumatic memories through non-verbal and embodied processes [10,11].

As a result, art therapy has been increasingly integrated into clinical pathways addressing various conditions, including depression, anxiety, personality disorders [11,12] trauma-related disorders, and eating disorders (EDs) [13], where it offers unique opportunities for emotional expression, identity reconstruction, and the processing of complex feelings that are often difficult to articulate verbally [1,9,13,14]. This complexity allows for a wide array of interventions, making art therapy particularly effective for addressing cognitive impairments, enhancing self-esteem, fostering emotional resilience, and improving social skills [4]. In terms of media, art therapy is flexible and encompasses diverse forms, including drawing, painting, drama, music, writing, and even digital platforms, all under the guidance of trained therapists [15]. This flexibility has facilitated its successful application across various mental illnesses, including schizophrenia [5], mood disorders, and trauma-related conditions [6]. In individuals with EDs, art therapy has also demonstrated benefits in enhancing treatment adherence and overall psychological well-being [7].

EDs such as anorexia nervosa (AN) and bulimia nervosa (BN) are severe psychiatric conditions with distinct clinical features. AN is characterized by persistent restriction of caloric intake, significant weight loss, distorted body image, and often excessive physical activity. Approximately 90% of individuals affected are female [8], and AN carries a high mortality risk due to complications from starvation [9]. BN, typically emerging during adolescence, affects up to 3% of females and more than 1% of males during their lifetime [16]. BN is marked by recurrent episodes of binge eating, accompanied by feelings of loss of control, followed by compensatory behaviors such as self-induced vomiting, laxative abuse, fasting, or excessive exercise [4]. These behaviors occur at least once per week for three months, and self-evaluation is heavily influenced by body shape and weight [4]. Both disorders profoundly affect physical and mental health, significantly reducing quality of life [8].

Effective management of EDs requires a comprehensive, multidisciplinary approach combining phased psychological interventions, nutritional rehabilitation, and pharmacotherapy when necessary [16]. Despite these structured treatment pathways, maintaining long-term remission remains a significant challenge [17]. High dropout rates, often linked to stigma, denial, and fear of change—particularly in inpatient settings—highlight the critical need for more patient-centered and acceptance-focused therapies [18]. Art therapy has emerged as a promising intervention capable of circumventing traditional barriers to treatment by facilitating non-verbal expression and reducing the reliance on over-rationalization, which often hinders verbal therapies in ED populations [19].

Comorbid anxiety disorders are common in individuals with EDs, further complicating treatment, worsening symptom severity, and contributing to poorer long-term outcomes [16]. Art therapy has shown potential in addressing these challenges by enhancing emotional expression and regulation, which are key therapeutic targets in both EDs and anxiety-related conditions [16,20]. Beyond its role in managing anxiety, art therapy may offer a range of additional benefits aligned with the complex psychological and interpersonal needs of individuals with EDs.

First, art therapy facilitates non-verbal expression, offering an alternative mode of communication for individuals who struggle to articulate emotions verbally—a common feature in ED populations often characterized by alexithymia, emotional avoidance, and difficulty recognizing internal states [21,22]. Through symbolic and sensory-based creative processes, art therapy helps externalize feelings and conflicts that may be inaccessible or defended against in traditional talk therapies [23,24].

Second, art therapy provides a safe, contained space to explore the body through symbolic and metaphorical means, which is particularly relevant given the pervasive body image disturbances and somatic dissociation present in EDs [25,26]. Creative engagement with materials—particularly textiles, which evoke bodily sensations and metaphors of containment, healing, and transformation—supports reconnection with the body in a gradual, non-threatening manner [27].

Third, art therapy promotes the integration of fragmented self-experiences. Many individuals with EDs experience identity disturbances, perfectionism, and internalized critical voices. Artistic processes enable the exploration of self-narratives, offering opportunities to reconstruct a more cohesive and compassionate sense of self through symbolic work, autobiographical themes, and the transformative act of creating with materials [24].

Fourth, art therapy supports relational healing within group contexts. In group settings, art therapy brings up interpersonal connection, shared experiences, and mutual recognition among participants, counteracting the social isolation and relational difficulties frequently associated with EDs. The co-creation of shared projects or reflective discussions around artworks enhances empathy, validation, and social belonging, all of which support recovery [28].

Finally, emerging research suggests that art therapy may positively influence neurobiological processes related to stress, emotional regulation, and self-awareness, such as reducing cortisol levels and engaging brain regions implicated in affective processing and self-perception. This may offer a unique complement to more cognitively focused interventions [17,29,30].

Understanding the distinct therapeutic needs for state versus trait anxiety—state anxiety being a temporary response to specific situations, while trait anxiety reflects a chronic predisposition toward heightened anxiety—is crucial [31]. While short-term interventions may alleviate acute anxiety symptoms [32,33], addressing trait anxiety requires sustained therapeutic engagement to promote lasting emotional transformation [34]. However, few studies have specifically investigated the short- and medium-term impacts of art therapy on both state and trait anxiety in adolescents with EDs using repeated-measures, within-session designs.

While the therapeutic potential of art-based interventions is increasingly acknowledged, their structured application within real-world clinical settings, such as residential programs for EDs, remains under-researched. Moreover, the nuanced distinction between the immediate and cumulative effects of art therapy on anxiety, particularly in adolescent populations, is seldom addressed in the literature [35].

The aim of this pilot study was to assess the short-term effects of a structured, textile-based art therapy program on state and trait anxiety levels in adolescent females diagnosed with AN and BN. By addressing this gap, the study seeks to contribute new insights into how art therapy may enhance comprehensive ED treatment strategies, potentially improving outcomes through greater emotional engagement and treatment adherence.

## 2. Materials and Methods

**Figure 1:**
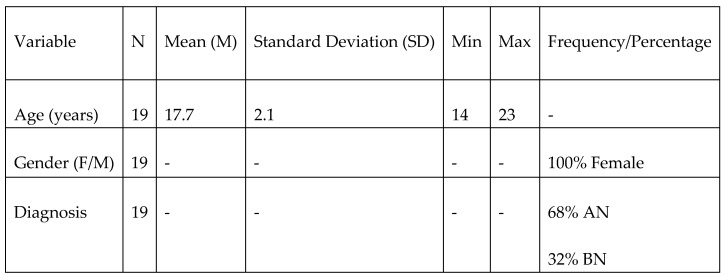
Descriptive samples analysis.

Nineteen female adolescents (average age 17.7 years ± 2.1) diagnosed with EDs (13 AN, 6 BN) and admitted to the Mariconda Regional Residence for Eating Disorders of the ASL of Salerno in residential or semi-residential regimes participated in this study (Figure 1). Participants were not receiving concurrent psychopharmacological treatment or structured cognitive-behavioral therapy (CBT) during the intervention period; they followed only the standard nutritional counseling program provided within the Center. Inclusion criteria required a formal diagnosis based on DSM-5-TR ED criteria and informed consent. Exclusion criteria included severe cognitive impairment or acute psychiatric decompensation.

### 2.1. Procedure

Participants underwent six art therapy sessions over a three-week period. Each session lasted 60 min and was conducted in a small-group format under the supervision of a certified art therapist. Before (T0) and after (T1) each session, participants completed the State-Trait Anxiety Inventory (STAI-Y1 for state anxiety, STAI-Y2 for trait anxiety [36] Spielberger, 1983). To minimize potential bias, the art therapist conducting the sessions was not involved in administering or scoring the anxiety measures, which were completed independently by participants under the supervision of a separate clinical staff member.

### 2.2. Description of the Art Therapy Intervention

#### 2.2.1. Art Therapy Intervention Description for Replication

Participants were enrolled from January to May 2025 and engaged in a structured six-week cycle of weekly art therapy sessions, each lasting approximately 60 min and facilitated by a qualified art therapist specializing in textile-based expressive arts. Sessions took place within a dedicated workshop space designed to provide a safe and supportive environment for creative exploration of body image, emotions, and personal history through symbolic and embodied artistic practices.

#### 2.2.2. Structure of the Sessions

Each session followed a consistent three-part format:

1. **Opening Phase**: Body-centered or symbolic activities were used to promote grounding and facilitate connection between bodily sensations and emotions.
2. **Art-Making Phase**: Creative activities were inspired by the work of contemporary female artists and focused on the use of textile materials to encourage symbolic expression.
3. **Closing Phase**: A group sharing circle allowed participants to reflect on their experience and process emerging emotions within a supportive peer context.

#### 2.2.3. Rationale for Materials and Techniques

Textile materials were selected for their accessibility, sensory qualities, and strong metaphorical resonance, symbolizing the human body’s fragility, resilience, and potential for transformation. Participants used techniques such as stitching, knotting, embroidering, tearing, and reassembling to support processes of mind–body reconnection, emotional expression, and narrative reconstruction. The intervention adopted an experiential and process-oriented pedagogical approach inspired by learning-by-doing principles and the atelier method, emphasizing symbolic exploration, hands-on engagement with materials, and self-directed meaning-making rather than technical instruction.

#### 2.2.4. Progressive Experiential Content of the Sessions

- **Session 1**: Participants embroidered a word onto fabric, chosen to represent an aspect of their bodily awareness identified during the opening exercise.
- **Session 2**: Drawing inspiration from *Women Who Run with the Wolves*, participants created symbolic “shelter spaces” using collage techniques and recycled fabrics.
- **Session 3**: A symbolic self-portrait was developed through botanical printing methods on fabric, encouraging reflection on personal identity and embodiment.
- **Session 4**: Circular mandala compositions were created using tulle, exploring themes of balance, transparency, and interconnectedness. These works were later projected as light installations within the workshop space.
- **Session 5**: Participants engaged in a reparative exercise, mending a torn canvas with colorful threads, referencing the Japanese philosophy of *kintsugi*, which values visible repairs as a metaphor for healing and integration of past wounds.
- **Session 6**: Each participant produced a final textile fragment incorporating key words and images from their personal journey. These fragments were collectively assembled into a large-scale textile manifesto, which was presented in a public exhibition.

#### 2.2.5. Closure and Reflection

The final exhibition served both as a closure ritual for participants and as a public reflection on the transformative potential of textile-based art therapy. Throughout the process, the textile medium functioned as a symbolic surface for processes of self-reconnection, emotional expression, and the rewriting of personal narratives.

### 2.3. Measures

State-Trait Anxiety Inventory (STAI): This validated self-report instrument comprises two subscales: state anxiety (STAI-Y1) and trait anxiety (STAI-Y2). Each subscale includes 20 items rated on a 4-point Likert scale, with higher scores indicating greater anxiety.

### 2.4. Statistical Analysis

Repeated-measures ANOVA was used to assess changes in state and trait anxiety across sessions and time points (pre vs. post). For trait anxiety, which was hypothesized to vary more gradually, a linear mixed-effects model was also applied, with time, session, and their interaction as fixed effects. No missing data were observed in the dataset, as all participants completed the anxiety assessments at each time point. All statistical analyses were performed using IBM SPSS Statistics for Windows, version 30 (IBM Corp., Armonk, NY, USA).

## 3. Results

### 3.1. State Anxiety

**Figure 2:**
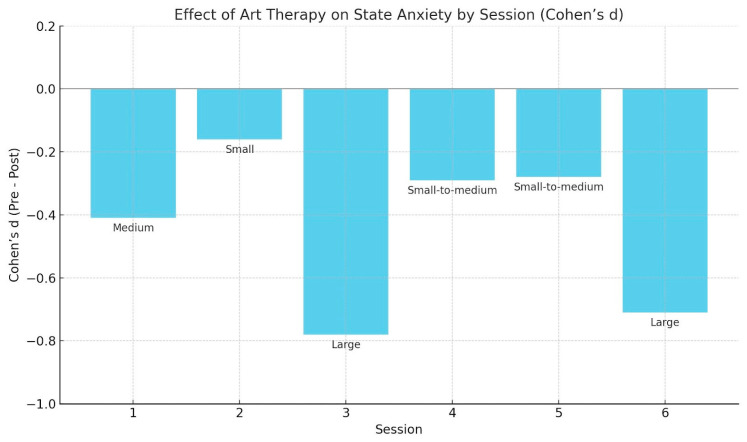
Art therapy effect on state anxiety.

A two-way repeated-measures ANOVA was conducted to examine the effect of an art therapy intervention on state anxiety, with time (pre vs. post) and session (1 to 6) as within-subject factors. The analysis revealed a significant main effect of time, F(1,18) = 12.5, *p* = 0.002, *η*^2^ = 0.16, indicating a general reduction in state anxiety after the sessions. There was also a significant main effect of session, F(5,90) = 3.2, *p* = 0.01, and a significant time × session interaction, F(5,90) = 2.8, *p* = 0.025, suggesting that the extent of anxiety reduction varied across sessions. To quantify the magnitude of change, Cohen’s d was calculated for the overall pre/post difference and for each session individually. The aggregated pre/post comparison across all sessions yielded a medium effect, d = −0.44, supporting the intervention’s effectiveness in reducing state anxiety. Cohen’s d values indicate the standardized mean difference between pre- and post-session state anxiety scores (Figure 2). Negative values reflect a reduction in anxiety following the session (Table 1).

### 3.2. Trait Anxiety

A two-way repeated-measures ANOVA was conducted to examine the effect of an art therapy intervention on trait anxiety, with time (pre vs. post) and session (1 to 6) as within-subject factors. The analysis revealed no significant main effect of time (post vs. pre-session: coefficient = −2.00, *p* = 0.11, *η*^2^ = 0.05), indicating no immediate reduction in trait anxiety after the sessions.

However, significant main effects of session were observed:

Session 4: β = −3.06, *p* = 0.015
Session 5: β = −6.11, *p* < 0.001
Session 6: β = −3.56, *p* = 0.005

These findings suggest a progressive reduction in baseline trait anxiety levels across the course of the intervention.

**Figure 3:**
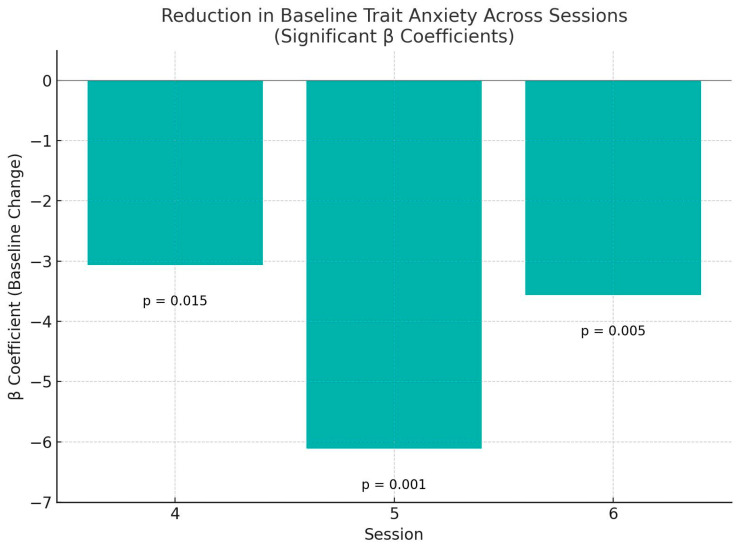
Trait anxiety reduction by session.

No significant time × session interaction was found (all *p* > 0.10), indicating that the pre/post difference remained relatively stable across sessions. The estimated effect size for session was medium (*η*^2^ ≈ 0.10), suggesting that a moderate proportion of the variance in trait anxiety scores was accounted for by session progression. To quantify the magnitude of change, Cohen’s d was calculated for the overall pre/post difference and for each session individually. The aggregated pre/post comparison across all sessions yielded a small-to-medium effect, d = −0.26, indicating a modest reduction in trait anxiety across the intervention. Cohen’s d values reflect the standardized mean difference between pre- and post-session trait anxiety scores (Figure 3).

## 4. Discussion

The findings of this study suggest that art therapy can serve as an effective adjunctive intervention for reducing both state and trait anxiety in adolescents with EDs. The observed significant decrease in state anxiety indicates that art therapy may be particularly useful for managing acute, situational distress commonly experienced by this population. These results align with prior research demonstrating that creative arts interventions—such as drawing, painting, and other expressive modalities—can rapidly alleviate transient anxiety symptoms [17,18]. Such reductions are particularly clinically relevant, considering that anxiety exacerbates ED symptom severity, impairs motivation for treatment, and contributes to poorer outcomes [11]. The impact on trait anxiety, however, was more distinctive and appeared to require a longer duration of engagement to observe a significant change. Reductions in trait anxiety only reached statistical significance beginning from session 4, suggesting that a cumulative therapeutic effect may be necessary before more stable anxiety patterns begin to shift. This delayed response aligns with the conceptualization of trait anxiety as a more enduring disposition, requiring sustained engagement in emotionally integrative interventions to achieve measurable change. Given that trait anxiety is characterized as a stable predisposition to perceive situations as threatening [20], modifying this trait necessitates sustained therapeutic effort. Art therapy’s capacity to facilitate deep emotional processing and self-awareness—especially through non-verbal expression—may gradually influence broader anxiety vulnerabilities over time [31]. This dovetails with theoretical models proposing that repeated exposure to creative expression can enhance emotional resilience, reduce tendencies toward hyperarousal, and foster a sense of mastery, thus contributing to diminished trait anxiety [37]. The therapeutic mechanism underlying these effects likely involves several interconnected processes. First, art therapy provides a non-threatening platform for externalizing internal conflicts such as shame related to body image or emotional suppression, common features in EDs [15]. Externalization through art allows patients to observe and reinterpret their internal experiences, fostering a sense of agency and reducing avoidance behaviors. Second, engaging in creative activities activates neural pathways associated with positive emotional regulation, sensory integration, and self-efficacy [4,38]. This multisensory engagement may stimulate neuroplasticity and promote adaptive emotional responses that generalize beyond therapy sessions. Neuroimaging studies have shown that visual and creative art-making activates brain regions involved in emotion regulation, such as the ventromedial and dorsolateral prefrontal cortex (PFC), as well as limbic structures like the amygdala. Moreover, engagement in artistic expression has been associated with increased activity in the brain’s reward circuitry, including the ventral striatum and nucleus accumbens, suggesting a dopaminergic contribution to the therapeutic experience [23,24,39]. Moreover, the non-verbal and symbolic nature of art therapy is particularly advantageous for adolescents who struggle to articulate difficult emotions such as shame, guilt, or trauma [40]. When integrated with traditional verbal therapies, art therapy can enhance emotional expression, strengthen the therapeutic alliance, and promote stabilization [41,42]. This complementary channel of communication increases treatment acceptability and engagement, particularly in individuals with eating disorders, where emotional avoidance and verbal inhibition are common [43,44,45]. Their synergistic use may produce more comprehensive improvements in psychological functioning. Despite these promising findings, several limitations deserve consideration. The relatively short follow-up period constraints conclusions about the durability of anxiety reductions, particularly for trait anxiety. Longer-term studies are needed to determine whether these benefits persist and how they influence ED symptom trajectories and treatment adherence over time. Furthermore, individual differences—including severity of illness, comorbidities, and personality traits—may influence responsiveness to art therapy. Identifying predictors of favorable response could allow for more personalized intervention plans. Another limitation relates to methodological variability; future research should aim to standardize intervention protocols and assess which specific artistic modalities—or combinations therein—are most effective for particular symptom profiles. Exploring the mechanisms of change through neuroimaging, psychophysiological measures, or qualitative assessments could deepen understanding of how art therapy exerts its effects. For example, studies could examine whether any neurobiological correlates of emotional regulation are modulated after art therapy sessions [46]. Clinically, these findings underscore the importance of incorporating art therapy into multimodal treatment frameworks for adolescent EDs, especially considering its accessibility, acceptability, and potential to improve engagement. Given that high dropout rates and treatment resistance remain challenges in this population [47,48], arts-based interventions may serve as motivating, low-stigma options that resonate with young patients’ developmental and emotional needs.

### Limitations and Future Directions

This study faces several limitations that should be addressed in future research to strengthen the evidence base for art therapy in adolescents with EDs. First, the small sample size limits the generalizability of the findings and reduces statistical power, making it difficult to draw definitive conclusions about the efficacy of art therapy across diverse populations. The use of a linear mixed-effects model on a relatively small sample (N = 19) may have limited the statistical power to detect subtle changes, particularly in trait anxiety. Larger multicenter studies are warranted to confirm these preliminary results and to explore potential moderators of treatment response, such as age, gender, ED subtype, and comorbid psychological conditions.

Second, the absence of a control group constrains the ability to attribute observed improvements solely to the art therapy intervention. Without a comparison condition such as standard psychotherapy, another expressive modality, or a waitlist control, it remains possible that nonspecific factors, including the natural course of recovery, placebo effects, or concurrent treatments, influenced the outcomes. As this was an uncontrolled, single-group pilot study, the observed improvements cannot be conclusively attributed to the art therapy intervention alone. The lack of a comparison group limits causal inference, and findings should be interpreted with caution when generalizing to broader populations. Future studies should employ randomized controlled trial (RCT) designs to rigorously isolate the specific effects of art therapy and to control for confounding variables.

Third, the study’s short follow-up period limits insights into the sustainability of the treatment effects. While initial reductions in both state and trait anxiety are promising, it remains unclear whether these benefits persist over time or translate into improved long-term recovery, treatment adherence, and reduced relapse rates. Incorporating longitudinal assessments at multiple follow-up points such as 6 months or 1 year post-intervention would provide valuable information regarding the durability of therapeutic gains and help identify factors supportive of sustained improvement.

Additionally, examining the mechanisms of change remains a crucial future direction. Investigations into how art therapy influences emotional regulation, self-esteem, body image, and other psychological constructs could elucidate its therapeutic pathways. Combining quantitative measures with qualitative data such as patient interviews and process analyses may reveal personalized aspects of engagement and response, informing tailored interventions.

Moreover, exploring the dose–response relationship, such as the optimal frequency and duration of art therapy sessions, would enhance clinical guidelines. It could also be beneficial to compare different artistic modalities (e.g., visual arts, dance, drama) to determine which are most effective for particular symptom profiles or anxiety dimensions.

## 5. Conclusions

This pilot study provides preliminary evidence that art therapy can serve as a valuable adjunctive intervention for reducing both state and trait anxiety in adolescents with EDs. The observed significant decrease in state anxiety suggests that art therapy is particularly effective in addressing acute, situational distress, a common challenge in this population. In contrast, the reduction in trait anxiety appeared more gradual, becoming statistically significant only from session four onward. This pattern supports the hypothesis that cumulative exposure to emotionally integrative and embodied therapeutic practices is necessary to affect more stable anxiety traits.

A key innovation of this study lies in the use of a textile-based art therapy protocol—an underexplored medium within ED treatment. Textiles were chosen for their symbolic resonance with the body’s fragility and potential for repair, offering participants a concrete, metaphorical means of processing emotions and bodily experiences through techniques such as stitching, tearing, and mending. The progressive experiential structure of the sessions further supported participants’ emotional and narrative reconstruction processes.

Moreover, this study contributes methodologically by employing a repeated-measures, within-session design to track both state and trait anxiety changes over time. This approach allowed us to capture not only immediate but also cumulative therapeutic effects, an area often overlooked in previous research.

Finally, the integration of this structured art therapy program within a real-world, residential ED treatment setting demonstrates the feasibility and clinical relevance of creative, body-based interventions in supporting emotional regulation, enhancing treatment engagement, and addressing longstanding therapeutic challenges such as high dropout rates.

## Figures and Tables

**Table 1:** Effect sizes by session.

Session	Cohen’s d	Effect Size Interpretation
Session 1	−0.41	Medium
Session 2	−0.16	Small
Session 3	−0.78	Large
Session 4	−0.29	Small-to-medium
Session 5	−0.28	Small-to-medium
Session 6	−0.71	Large

## Data Availability

De-identified data may be made available from the corresponding author upon reasonable request. The analysis code used in this study is available upon request from the corresponding author.
